# Materia Medica and Therapeutics; Medical Chemistry; Toxicology and Legal Medicine

**Published:** 1860-09

**Authors:** Emil Fischer


### MATERIA MEDICA AND THERAPEUTICS; MEDICAL CHEMISTRY; TOXICOLOGY AND LEGAL MEDICINE.

BY EMIL FISCHER, M.D.

### MATERIA MEDICA AND THERAPEUTICS.

I.—*Kamela: (Rottlera Tinctoria;) its History, Properties, Medical Uses, Doses, etc.*

*History.*—This plant was known to the Hindoos from the remotest antiquity, the fruit of which was employed by them as a remedy for worms, and also in certain skin diseases. They likewise used it for dyeing and printing silks, etc., but for this purpose it underwent a chemical process, viz., an alkaline solution was mixed with the powder of the fruit, which yielded a beautiful orange-brown color. But its properties have only recently been demonstrated by Drs. Anderson and Mackinnon, who first introduced it into England. Kamela belongs to the natural order *Euphorbiaceæ.* It grows abundantly in the hilly districts of India, Burmah, the Philippine Islands, and the northeast portions of Australia. Its fruit ripens in February and March, when it is gathered, and the powder, which appears as an excrescence, is carefully brushed off, and preserved for use.

*Properties.*—The powder of kamela is of a red-brick color, with little or no oder and flavor, and, like lycopodium, it is difficultly miscible with water. Ether extracts a quantity of its resinous components, and if the solution be allowed to stand for a few days a precipitate of granular crystals is formed, which, when purified, is seen to consist of small scales of a yellowish color and satiny lustre, and named, by Dr. Anderson, rottlerine. Alcohol extracts its medicinal constituents most perfectly, and the tincture made with rectified spirit is found to be very suitable for administration. Its formula, on analysis, is found to be $C^{22}H^{10}O^{6}$. Its composition is as follows:—

| | |
|---|---|
| Resinous coloring matter | 78·19 |
| Albuminous substances | 7·34 |
| Cellulose, etc | 7·12 |
| Carbon | 3·86 |
| Water | 3·49 |
| Volatile oil | A trace. |
| Volatile coloring matter | A trace. |
| | 100·00 |

*Medical uses.*—Kamela has been successfully given in cases of tænia, and Dr. Mackinnon considers it of much greater value in those cases than either kousso or turpentine. Dr. Anderson describes ninety-five cases of tænia, in "ninety-three of which the worms were expelled after the third or fourth dose." It has been used, also, with very great success for tape-worm and ascarides, by Dr. Arthur Leared, of the Great Northern Hospital, and by Dr. William Moore, of Dublin. The *Lancet*, of May 15th, 1858, records six cases of cure of tape-worm, by Dr. Ramskill, of the Royal Free Hospital. In some instances sickness, headache, and purging are produced by it, but generally its administration is attended by no unpleasant result.

*Dose.*—In powder, the dose of this remedy is from one to three drachms early in a morning, fasting. The tincture (*made by macerating half a pound of powder of kamela for fourteen days in a pint of rectified spirits of wine*) may be given in doses of from one to two drachms, night and morning, on lump sugar.—(*Chemist and Druggist*, London, March 19, 1860.)

II.—*On the Use of Chloride of Zinc in Diseases of the Skin.* By Dr. Veiel.

Dr. Veiel applies chloride of zinc, in the hospital at Cannstatt, in three different forms, viz., in alcoholic and in aqueous solution, and in the solid form, as stick or cylinder. The first of these preparations consists of equal parts of alcohol and chloride of zinc; the second, of ten parts of chloride of zinc, ten parts of muriatic acid, and five hundred parts of water; the third is obtained by heating chloride of zinc and casting it into sticks. The solid form (lapis zinci chloridi) the author makes use of in cases where he wants to penetrate to the greatest possible depth in order to destroy hypertrophic deposits, as are often seen in cases of *lupus hypertrophicus* and *tuberculosus;* in these cases the chloride of zinc is applied in exactly the same manner as caustic potassa according to the directions of Langenbeck. In *lupus exfoliatus* and *exulcerans* without hypertrophic infiltration, the spiritus zinci chloridi answers the purpose, and is used until the more or less developed or already softened tubercles are completely destroyed; the cure is then completed with the aqueous solution, (liquor zinci chloridi,) which is used also after the application of the stick. *Lupus superficialis sive erythematosus* requires the spiritus zinci chloridi, diluted with the liquor, for its treatment, as in this form of the disease the more superficial layers of the skin are implicated, and the morbid process rarely extends to the subcutaneous cellular tissue.

The indications for the three different forms of chloride of zinc are, in short, the following: The stick is to be applied in all cases where hypertrophic tissues require to be removed; the spiritus, where diseased subcutaneous tissues and degenerated superficial layers of skin are to be destroyed; the liquor, where the cure is to be hastened by an astringent and stimulating remedy, and a firm cicatrix is to be obtained. Besides in lupus, chloride of zinc is likewise applicable to many other cutaneous diseases: as for instance, to obstinate eczema, (the spiritus;) to remnants of psoriasis as are left after a cure by the preparations of tar, (the spiritus;) to sycosis, and favus after the removal of the hair, (the liquor;) to wart-like, circumscribed, scirrhous tumors on the nose, the cheeks, or lips, (spiritus;) to chronic, particularly callous ulcers of the leg, (spiritus;) to cysts, follicular tumors, fistulous passages, (spiritus;) to condylomata, soft warts, molluscum, (lapis,) seborrhœa, burns, and chilblains, (liquor.)

The principal advantages of chloride of zinc over other caustics are the following: 1. Chloride of zinc forms chemical compounds with all organic elements with which it comes into contact, especially with proteinaceous and albuminous substances; and those compounds themselves produce a deeply penetrating, caustic, and stimulating effect, in consequence of which the parts in their immediate neighborhood contract; the wound is thus rendered smaller, and the sound parts are brought nearer together. 2. The same stimulating effect hastens the establishment of suppuration and the detachment of slough; the healing process is thus accelerated, the surface of the wound assumes a more lively appearance, and exhibits better granulations. 3. In consequence of the contraction and uniform destruction, a superior cicatrix is formed. 4. The pain, although considerable in the beginning, lasts a comparatively short time, and can therefore be moderated very much by the administration of chloroform. By these properties chloride of zinc surpasses, first, the acids which produce more coagulation of the albuminous substances, and form a tough, leathery eschar, which does not penetrate very deeply and detaches itself but slowly and with difficulty, as the shrinking of the capillary vessels has an injurious effect upon the healing process; second, caustic potassa, which causes a saponification of the soft parts, not easily confined to proper limits, corrodes the surrounding tissues by its secretion, and produces eschars which are also slow in detaching themselves, and leave neither a favorable suppuration nor vigorous granulations behind; third, nitrate of silver, which acts much more superficially, establishes a more tardy suppuration of the destroyed tissues, and therefore causes more protracted pain and an inferior cicatrix; fourth, the preparations of iodine (the iodurated glycerin of Richter, and the preparations of mercurial iodides) penetrate, likewise, with less rapidity, produce a greater reaction of the surrounding parts, are therefore more painful, and, on account of their variable composition, uncertain in their action. With chloride of gold, chloride of bromine, Landolfi's paste, Pétrequin's caustic solution of gold in nitromuriatic acid, the author has not made any comparative experiments; nor with Cosme's powder or other arsenical preparations, which he has avoided on account of their dangerous and unreliable character.—(*Zeitschrift der K. K. Gesellschaft der Aerzte zu Wien*, iii. 8, 1860, and *Schmidt's Jahrbücher*, cvi., May, 1860.)

III.—*On the Efficacy of some Antipyretic Remedies.* By PROF. W. VOGT.

In a very extensive treatise on the antipyretic method of treatment, and its use in acute diseases in general, the author discusses the relative value of the different antipyretic remedies. In regard to the most important of these, *quinine* and *veratrine*, the author's experience seems to show that their power of suppressing fever appears to be nearly equal, if not somewhat stronger in veratrine. The action of veratrine seems to be more direct; it takes place without previous excitement, and proceeds principally from the spinal marrow. That of quinine, on the contrary, emanates, it seems, from the brain, and is preceded by excitement. It is more indirect, and rather a secondary than a primary effect. Hence it follows that veratrine probably deserves the preference in febrile diseases which are violent and rapid in their course. The excellent results which the author has observed from the administration of veratrine in pneumonia, induced him to abandon the use of quinine in the treatment of this disease. From this highly beneficial effect of veratrine, the author was led to suppose that it would be equally serviceable in puerperal fevers of a malignant and violent character. In typhoid fever, it is to be preferred at the commencement of the disease; so much the more the higher the fever is, the greater a general hyperæmia, and the stronger and more active congestions of the brain are present. In cases taking a slower course, if the febrile reaction is less violent, if the hyperæmia is owing to venous stagnation, and, finally, in the later periods of the fever, where an anæmic and adynamic condition is observable, quinine is to be preferred. Veratrine has a stronger antipyretic effect than quinine. When the eruptive fevers show a tendency to real local inflammations, veratrine deserves the preference. The author arrived at this conclusion from its favorable action in pneumonia and those puerperal fevers which are distinguished by intense local inflammation. If the fever appears to be, however, more independent, and not accompanied by any essential local inflammation, quinine is preferable, provided that the character of the case does not present decided indications for the use of veratrine. The toxical effects of quinine are more serious, and cannot be watched as easily as those of veratrine. In typhoid fevers it was unmistakable that veratrine did more toward relieving the head from unpleasant symptoms than quinine. The latter admits of the doubt whether, after its exhibition, the cerebral affection is owing to the disease or to a remaining intoxication, produced by quinine. If the cerebral symptoms are caused more by venous congestion, quinine may act more favorably than veratrine. The author prefers, consequently, veratrine as a general antipyretic; in regard to other effects, however, quinine surpasses veratrine in malarious fevers of a less rapid course, in other febrile diseases during the later periods, where symptoms of anæmia and adynamia have commenced to show themselves, and where the fever is to be slowly suppressed.

Among other antipyretic remedies recently used, the author mentions *digitalis* and the *alcoholic remedies*, which were employed already by Brown. He has convinced himself of the powerful antipyretic properties of digitalis in a small number of cases of pneumonia, but was deterred from further experiments by the dangerous symptoms produced by the drug. The author has not tried the method proposed by Todd, according to which fever and inflammations are treated by larger doses of alcoholic remedies.—(*Schweizer Mon. Schr.*, iv. 5, 6, 7, and *Schmidt's Jahrbücher*, 2, 1860.)

IV.—*Tinctura Thuiæ in bad Smells of the Nose.* By DR. HOPPE.

Dr. Hoppe says that of all means for correcting bad smells of the nose, usually dependent upon excoriations of the mucous membrane, the *tinctura thuiæ*, (American *arbor vitæ*,) applied upon the finger, is the best, and although only a palliative, it is a very effectual one in a very annoying infirmity. It is also of use in diminishing the smell arising from smegma præputii, and the smells proceeding from the feet and axilla.—(*Berlin Med. Zeitung*, 1859, No. 52, and *Med. Times and Gazette*, March 31, 1860.)

V.—*Chlorodyne; its History, Preparation, Properties, Therapeutic Effects, Doses, etc.*

*History.*—Chlorodyne was invented in the year 1848 by Dr. Browne while officiating in his medical capacity during the prevalence of cholera and diarrhœa among the troops in India, and was introduced to the notice of the faculty by him as "a combination of perchloric acid with a new alkaloid."

*Preparation.*—From Dr. Ogden's analysis it appears to be composed as follows:—chloroform, six drachms; tincture of capsicum, half a drachm; oil of peppermint, three drops; muriate of morphia, eight grains; perchloric acid, twenty drops; Steele's hydrocyanic acid, twelve drops; tincture of Indian hemp, one drachm; treacle, one drachm. Dissolve the morphia in the perchloric acid; then add the tincture of hemp, capsicum, peppermint, and chloroform, and lastly, the treacle and prussic acid.

*Properties.*—Chlorodyne is a volatile liquid possessing a pungent smell and taste. It is soluble in alcohol, but insoluble in water; but may be conveniently administered in that liquid by suspending it in a little mucilage. The alkalies and alkaline salts decompose it. In color it is dark brown, and in weight equal to twice its bulk of water. It is anodyne, sedative, diaphoretic, astringent, antispasmodic, diuretic, etc. Unlike the preparations of opium, it does not produce headache, giddiness, prostration of strength, nor stupor; but in large doses, and from a constipated state of the bowels, it is liable to produce nausea, which in the former case may be relieved by a small dose of sal-volatile, and in the latter by recourse to aperients.

*Therapeutic effects.*—The changes produced by this preparation on the system, are—first, a gentle heat at the stomach, followed by a general glow and total absence of pain; second, a calm and refreshing sleep; and, third, an increase in the pulse from a "small, weak, thready, hurried, or bounding one to a full, yielding, elastic, natural sort of one, decreasing in frequency of beats as well as resistance to a healthy condition."

Of its powers in the cure of consumption, Dr. Stonehouse remarks: "The cases (among others) in which I have employed it have been twelve cases of phthisis; eight of these patients had been examined by other medical men, and had been regarded as genuine cases of consumption, so that the nature of the disease does not rest upon my testimony alone. They were all well-marked cases: for I do not mention several others in an incipient stage. Two of the cases were in the last stage—*i e.* cavities had formed in the lungs; two others were bordering upon this stage. The remaining eight were in the second stage, that of softening; in five of these hæmoptysis was a prominent symptom. All these cases have done or are doing exceedingly well. Five of them have quite recovered; the others, with one exception, are in a fair way toward recovery."

*Doses.*—The dose of this preparation must be regulated according to the nature of the complaint. As an anodyne for febrile, inflammatory, or neuralgic affections, the dose is from ten to thirty drops; diaphoretic, in cases of coughs, colds, etc., ten to twenty drops; sedative, in consumption, etc., twenty to fifty drops; antispasmodic, in gout, rheumatism, etc., twenty to forty drops; astringent, in cholera, diarrhœa, etc., fifty to one hundred drops. It is best administered on lump sugar, and given at intervals from every half hour to every four hours.—(*Chemist and Druggist*, February 15, 1860.)

VI.—*On Opiated Colchicum Wine in Rheumatism.* By DR. EISENMANN.

Dr. Eisenmann, of Würzburg, first states the wide application which he gives to the word "Rheumatism," denoting by it every affection which may arise in the healthy system, independently of any specific cause, from exposure to cold. "By exposure to cold I do not understand merely the effect produced by the contact of cold water or cold and humid air with the external integument, but also that which takes place when cold and damp air penetrates into the lungs, or when cold water is taken into the stomach, the temperature of the body having been raised by exercise." This view is justified by the facts—1. That cold gives rise to the most various affections of the nervous and vascular systems. 2. That these various affections may become, by metastasis, transformed into each other. 3. That they communicate to the economy a marked predisposition to affections of the same nature, such predisposition being increased with each reproduction. 4. That they yield to the same treatment, whether they show themselves under the form of neuroses or of vascular affections. Under the title of rheumatic inflammation, therefore, the author ranges all inflammatory affections of the heart, lungs, pleura, peritoneum, kidneys, serous membrane of the liver, etc., when these are not due to any specific cause, and treats them in the same manner as acute articular rheumatism.

The means which beyond all others he has found of efficacy in the treatment of rheumatism is a mixture of colchicum and opium, the colchicum acting far more efficaciously when so combined, and then not giving rise to the half-poisonous effects which often attend its use when given uncombined. Neither the one nor the other substance will produce alone the advantageous effects which result from their union. Dr. Eisenmann speaks not only from his own large experience, but from that of many of his medical friends, among whom "Eisenmann's Drops" have acquired a great reputation. These consist of twelve parts of colchicum wine and two of tincture of opium, twenty drops being taken three times a day. Instead of preparing the colchicum wine with sherry, as he formerly did, he now makes it according to the formula of the Prussian pharmacopœia, which directs one hundred and fifty parts of colchicum seeds to be macerated in seven hundred and seventy of alcohol; this preparation being always uniform in strength, and more active than the ordinary colchicum wine.

Although the above drops succeed so well in acute rheumatic affections, they are of little or no use in old and chronic cases. This induced the author to try the effect of adding minute doses of corrosive sublimate; and although his trials of this modification have been as yet too few to admit of an opinion being pronounced in respect to chronic cases, he has found the addition of great advantage in many cases of the acute form. Although in the various forms of rheumatism in which he has employed this treatment, he has not had to have recourse to preliminary bleeding, he by no means denies that this may not be occasionally desirable in the robust.

In treating *acute articular rheumatism* in this way, its course has usually been cut short in from the third to the fifth day, convalescence rapidly following, and no trace of heart affection persisting. When the pains have been very severe, tepid applications of a very weak solution of corrosive sublimate have been made to the joints, with the most satisfactory results. Sometimes after a rapid amelioration by means of the colchicum, when the pulse still continues irritable, and the tongue remains loaded, an emetic or purgative expedites convalescence. Among the rheumatic affections of the mucous membranes which may be rapidly and durably cured by means of the opiated colchicum, without the sublimate, may be specified angina, pulmonary catarrh, and influenza, gastric fever, catarrhal diarrhœa, and catarrho-rheumatic conjunctivitis. In the case of catarrhal ophthalmia, even of a severe character, its remarkable efficacy may be watched step by step. Among affections of the *serous membranes*, pleurisy and perihepatitis stand prominently forward as amenable to this treatment. Of parenchymatous inflammations, pneumonia has been the only one in which the medicine has been tried, and that only in two slight cases, which recovered with rapidity. In *muscular rheumatism* of the head, loins, etc., from two to four doses have always sufficed. It is also of great efficacy in cases of *rheumatic neuralgia*, especially in facial or intercostal, in sciatica and odontalgia. But the case must be recent, or it will be of no avail. In *odontalgia* the results are truly remarkable, a single dose rapidly dissipating the pain. The distinction between the rheumatic form of odontalgia and that arising from carious teeth is exhibited by the different effects of the colchicum. For the relief of odontalgia arising from carious teeth, the author, after having cleaned out the cavity of the tooth, introduces into it a morsel of nitrate of silver as large as a pin's head. In about a minute the moisture of the mouth dissolves this, and the mouth is then to be gargled with cold water, and the pain disappears. He has employed this plan of relieving the pain of carious teeth for the last twenty years, and he has seldom known it fail even after the ineffectual trial of various other measures. It causes no pain and it retards the progress of the caries.—(*Bulletin de Thérap.*, tome lvi. pp. 75, 120, and *Medical Times and Gazette*, April 14, 1860.)

VII.—*Caffein as an Antidote to the Poisonous Effects of Opium.* By Professor Henry F. Campbell.

Professor Henry Frazer Campbell, of Georgia, in the *Southern Medical and Surgical Journal* for May, 1860, publishes an article on this subject. After giving some account of caffein, and the use of coffee as an excitant, he describes the patient as a young man, twenty-four years old, who had, in a fit of mental depression, swallowed an ounce and a half of laudanum, nearly an hour before his visit, eight P.M. Narcotism had proceeded so far that emetics could not be employed; the muscles were greatly relaxed, the tongue falling back in the mouth and tending to stop respiration, and respiration exceedingly feeble. Cold water was applied to the head for a time, artificial respiration resorted to, and the stomach-pump then vigorously applied. At twelve o'clock P.M., the case seemed more hopeless; respirations but four to the minute. Artificial respiration in a sitting posture was then resorted to, which seemed to have a temporary good effect. Dr. Campbell then thought of coffee, but the patient could not swallow, and he doubted the propriety of using the stomach-tube in the then condition of the patient. Finding it difficult to get a strong infusion of coffee, the idea of employing caffein occurred; and having dissolved twenty grains in a quantity of infusion of coffee, it was administered as an injection by means of a syringe. In half an hour the respirations were eight per minute; an hour had not elapsed before he "*forcibly jerked his left arm from the assistants,*" and told them "to let him alone." The narcotism slowly passed off, and at ten o'clock next day, when Dr. Campbell called, the patient had left the hotel and gone home. Although the effects of the narcotism were apparent for several days, they soon wore off, and the patient entirely recovered. Dr. Campbell believes that the caffein in this case was largely concerned in the recovery, and points to that alkaloid as probably possessing valuable antidotal powers in cases of narcotic poisoning from this drug.—(*Am. Jour. of Pharmacy,* July, 1860.)

---

### Medical Chemistry.

*On the Method of Detecting the Presence and Estimating the Quantity of Sugar in Diabetic Urine, by means of Concentrated Sulphuric Acid.* By James M'Grie, M.D., Glasgow.

A considerable variety of methods have been given by different writers on urinary diseases, for testing diabetic urine, and estimating the quantity of sugar contained in any given specimen. The sense of taste is perhaps the readiest means of ascertaining that saccharine matter is present in urine, and evaporation gives an approximate estimate of the quantity. Trommer's test is now most commonly employed, and the improvements made upon it by Barreswill and Fehling enable us to employ it not merely for the qualitative, but also for the quantitative determination of sugar in the urine. The fermentation test, also, is one fitted to give satisfactory results.

Having been engaged in 1849 in the examination of diabetic urine, I had frequently tried all the tests usually employed, and among others that of Pittenkofer, with bile and sulphuric acid. At that time there was no notice of Runge's test in Dr. Golding Bird's work, though it appeared in a subsequent edition. Runge only contemplated the production of a blackish color by applying dilute sulphuric acid to diabetic urine; while Pittenkofer employed it along with bile to produce a violet color. Neither of these methods affords satisfactory results. "Grape sugar," says Thudichum, "is not influenced by dilute acids, and not blackened by concentrated sulphuric acid. If grape sugar, dried at a temperature of 100°, is mixed with one and a half times its weight of concentrated sulphuric acid, it dissolves, and forms with the acid a combination which is an acid itself, and has been called sulpho-saccharic acid." However just this observation may be in reference to dried grape sugar, it does not apply to that form of sugar found in diabetic urine. When concentrated sulphuric acid is added to an equal volume of diabetic urine, and heat applied to ebullition of the mixture, the following changes occur:—

First, the fluid becomes blackish, and soon passes into a state of inky blackness. As soon as ebullition commences, the mixture begins to froth up, and continues to be covered with a layer of froth so long as the boiling continues. This layer of froth also remains after the mixture is cooled. In no other kind of urine except the diabetic are these appearances manifested. After the boiling has been continued for a short time, besides the intense black color, when the fluid is closely examined, there will be found copious evolution of carbonaceous particles. The high specific gravity of the fluid in which these fine black particles are suspended prevents their being deposited as a precipitate till it is largely diluted with water. In ordinary samples of diabetic urine a copious precipitate is speedily deposited on the addition of water. The precipitate should now be thoroughly washed on a weighed filter, till all the acid is removed and nothing but the pure carbon remains on the filter. After being carefully dried and weighed, the excess of weight of the filter containing the carbon over the weight of the filter without the carbon, will give the amount of carbon contained in the sugar; and when this quantity is found it is easy to calculate the weight of the sugar. The dried precipitate on the filter will be found in the form of numerous small pieces of jet-black carbon, which may be collected, and, by combustion, will be dissipated in the form of gas, without leaving any solid residue.

It may be objected to this test, that there are other kinds of urine which, when boiled with sulphuric acid, develop appearances somewhat analogous, if not altogether identical with those produced by diabetic urine. It is true that albuminous urine, when boiled with strong sulphuric acid, gives a deep, rich-brown color, approaching to black; but it presents none of the peculiar phenomena which we have noticed as accompanying diabetic urine when treated in a similar manner. The color is different; it does not froth; and when diluted with water, there is no deposit of black, carbonaceous matter, but the deep-brown color merely becomes less deep in shade.

Any probable fallacy as between albuminous and diabetic urine, will, if the following method is adopted, be completely dissipated. Let the urine be first acidulated with $NO^5$, and then boiled, when, if albumen is present, it will become coagulated, and may be removed by filtration; then proceed with the test as described above.

I have seen this test fail to develop the characteristic phenomena when applied after the urine has been kept a sufficient length of time to admit of decomposition taking place. A sample of urine which has already been found by Trommer's or some other test to contain sugar, may, after it has stood some time, be tested by sulphuric acid, and will probably fail to produce any of the characteristic reactions. It will be found in all such cases that decomposition has begun, and not only this, but every other test will fail. The precaution ought to be taken to have the urine operated upon as fresh and recent as possible, when embarrassing failures of this kind will be avoided.

The *modus operandi* of the sulphuric acid in its action on grape sugar is extremely simple and well defined. The sugar consists of $C^{12}O^{12}H^{12}$; that is, twelve atoms of carbon are combined with twelve atoms of HO or water. Strong sulphuric acid has a sufficiently strong affinity for water to decompose the sugar and remove from it all the elements of HO, and leave the insoluble carbon disseminated in fine particles through the mixture; and whatever action it may have upon the other constituents of the urine, it does not give rise to any other insoluble compound, if the necessary precautions have been taken at the outset to remove the mucus by filtration, and the albumen, if there is any present, in the manner indicated above. In some specimens of high-colored and concentrated urine, when there is a considerable quantity of animal extractive and coloring matter, the action of the sulphuric acid may develop a small amount of blackish particles, which, when treated in the same way as the carbon of diabetic sugar, are easily distinguished from it. It may be affirmed, without fear of contradiction, that there is no other kind of urine which comports itself in the presence of sulphuric acid in a manner analogous to diabetic urine. Grape sugar, in the solid state, is dissolved in $SO^3$, but in solution in the urine is decomposed by it. When treated with caustic potash and heat, the smell of burnt sugar is evolved, the solution becomes brown in color, and glucic and molassic acids are produced. In the crystallized state it contains, besides the constitutional elements of water, two equivalents of water of crystallization, which are dispelled by a temperature of 212°. When the temperature is raised to 284°, three equivalents of constitutional water are dispelled, and caramel is produced; but it is only when in a state of solution that sulphuric acid removes all the constitutional elements of HO. Of the ordinary tests Trommer's is preferred by some, Cappezuoli's, and the fermentation test by others. The sulphuric acid test is one which, if deemed worthy of a trial, will, in point of simplicity in manipulation and certainty in its results, be found second to none.—(*Glasgow Medical Journal*, April, 1860.)

---

### TOXICOLOGY AND LEGAL MEDICINE.

1.—*On the Arsenic Eaters of Styria.* By CHARLES HEISCH, ESQ., F.C.S., Lecturer on Chemistry at the Middlesex Hospital Medical College.

"At the last meeting of the Manchester Philosophical Society, I observe that Dr. Roscoe called attention to the arsenic eaters of Styria. Having for the last two years been in communication with the medical men and other residents in the districts where this practice prevails, I shall feel obliged if you will allow me, through your journal, to make known the facts I have at present collected. The information is derived mainly from Dr. Lorenz, Imperial Professor of Natural History, formerly of Salzburg, from Dr. Carl Arbéle, Professor of Anatomy in Salzburg, and Dr. Kottowitz, of Neuhaus, besides several non-medical friends. If human testimony be worth anything, the fact of the existence of arsenic eaters is placed beyond a doubt. Dr. Lorenz, to whom questions were first addressed, at once stated that he was aware of the practice, but added, that it is generally difficult to get hold of individual cases, as the obtaining of arsenic without a doctor's certificate is contrary to law, and those who do so are very anxious to conceal the fact, particularly from medical men and priests. Dr. Lorenz was, however, well acquainted with one gentleman, an arsenic eater, with whom he kindly put me in communication, and to whom I shall refer again more particularly. He also says that he knows arsenic is commonly taken by the peasants in Styria, the Tyrol, and the Salzkammergut, principally by huntsmen and wood cutters, to improve their wind and prevent fatigue. He gives the following particulars :—

"The arsenic is taken pure in some warm liquid, as coffee, fasting, beginning with a bit the size of a pin's head, and increasing to that of a pea. The complexion and general appearance are much improved, and the parties using it seldom look so old as they really are; but he has never heard of any case in which it was used to improve personal beauty, though he cannot say that it is never so used. The first dose is always followed by slight symptoms of poisoning, such as burning pain in the stomach, and sickness, but not very severe. Once begun, it can only be left off by very gradually diminishing the daily dose, as a sudden cessation causes sickness, burning pains in the stomach, and other symptoms of poisoning, very speedily followed by death.

"As a rule, arsenic eaters are very long lived, and are peculiarly exempt from infectious diseases, fevers, etc.; but unless they gradually give up the practice, invariably die suddenly at last.

"In some arsenic works near Salzburg, with which he is acquainted, he says 'the only men who can stand the work for any time are those who swallow daily doses of arsenic, the fumes, etc. soon killing the others.' The director of these works, the gentleman before alluded to, sent me the following particulars of his own case. This gentleman's name I suppress, as he writes that he does not wish the only thing known about him in England to be the fact that he is an arsenic eater; but if any judicial inquiry should arise which might render positive evidence of arsenic eating necessary, his name and testimony will be forthcoming:—

"'At seventeen years of age, while studying assaying, I had much to do with arsenic, and was advised by my teacher, M. Bönsch, Professor of Chemistry and Mineralogy at Eisleben, to begin the habit of arsenic eating. I quote the precise words he addressed to me. "If you wish to continue the study of assaying, and become hereafter superintendent of a factory, more especially of an arsenic factory, in which position there are so few, and which is abandoned by so many, and to preserve yourself from the fumes which injure the lungs of most, if not of all, and continue to enjoy your customary health and spirits, and to attain a tolerably advanced age, I advise you, nay, it is absolute necessary, that besides strictly abstaining from spirituous liquors, you should learn to take arsenic : but do not forget, when you have attained the age of fifty years, gradually to decrease your dose till, from the dose to which you have become accustomed, you return to that with which you begun, or even less." I have made trial of my preceptor's prescriptions till now, the forty-fifth year of my age. The dose with which I began and that which I take at present, I inclose : they are taken once a day, early, in any warm liquid, such as coffee, but not in any spirituous liquors.' The doses sent were No. 1, original dose, three grains; No. 2, present dose, twenty-three grains of pure white arsenic in coarse powder. Dr. Arbéle says this gentleman's daily dose has been weighed there also, and found as above. Mr.—— continues : 'About an hour after taking my first dose, (I took the same quantity daily for three months,) there followed slight perspiration, with griping pains in the bowels, and after three or four hours a loose evacuation ; this was followed by a keen appetite, and a feeling of excitement. With the exception of the pain, the same symptoms follow every increase of the dose. I subjoin as a caution, that it is not advisable to begin arsenic eating before the age of twelve or after thirty years.' In reply to my question if any harm results from either interrupting, or altogether discontinuing the practice, he replies: 'Evil consequences only ensue from a long-continued interruption. From circumstances, I am often obliged to leave it off for two or three days, and I feel only slight languor and loss of appetite, and I resume taking the arsenic in somewhat smaller doses. On two occasions, at the earnest solicitations of my friends, I attempted entirely to leave off the arsenic. The second time was in January, 1855. I was induced to try it a second time, from a belief that my first illness might have arisen from some other cause. On the third day of the second week after leaving off the dose, I was attacked with faintness, depression of spirits, mental weakness, and a total loss of the little appetite I still had : sleep also entirely deserted me. On the fourth day I had violent palpitation of the heart, accompanied by profuse perspiration. Inflammation of the lungs followed, and I was laid up for nine weeks, the same as on the first occasion of leaving off the arsenic. Had I not been bled, I should most likely have died of apoplexy. As a restorative I resumed the arsenic eating in smaller doses, and with a firm determination never again to be seduced into leaving it off, except as originally directed by my preceptor. The results on both occasions were precisely the same, and death would certainly have ensued had I not resumed arsenic eating.' One of the most remarkable points in this narrative is that this gentleman began with a dose which we should consider poisonous. This is the only case of which I have been able to obtain such full particulars; but several others have been mentioned to me by those who knew the parties and can vouch for their truth, which I will briefly relate.

"One gentleman, besides stating that he is well aware of the existence of the practice, says he is well acquainted with a brewer in Klagenfurth, who has taken daily doses of arsenic for many years. He is now past middle life, but astonishes every one by his fresh juvenile appearance; he is always exhorting other people to follow his example, and says: 'See how strong and fresh I am, and what an advantage I have over you all! In times of epidemic fever or cholera, what a fright you are in, while I feel sure of never taking infection.'

"Dr. Arbéle writes: 'Mr. Curator Kursinger, (I presume curator of some museum at Salzburg,) notwithstanding his long professional work in Lungau and Brinzgau, knew only two arsenic eaters—one, the gentleman whose case has just been related, the other, the ranger of the hunting district in Grossarl, named Trauner. This man was, at the advanced age of eighty-one, still a keen chamois hunter, and an active climber of mountains; he met his death by a fall from a mountain height while engaged in his occupation. Mr. Kursinger says he always seemed very healthy, and every evening regularly, after remaining a little too long over his glass, he took a dose of arsenic, which enabled him to get up the next morning perfectly sober and quite bright. Professor Fenzl, of Vienna, was acquainted with this man, and made a statement before some learned society concerning him, a notice of which Mr. Kursinger saw in the *Wiener Zeitung*, but I have not been able to find the statement itself. Mr. Krum, the pharmaceutist here, tells me that there is in Sturzburg a well-known arsenic eater, Mr. Schmid, who now takes daily twelve and sometimes fifteen grains of arsenic. He began taking arsenic from curiosity, and appears very healthy, but always becomes sickly and falls away if he attempts to leave it off. The director of the arsenic factory, before alluded to, is also said to be very healthy, and not to look so old as forty-five, which he really is.' (The man above mentioned seems quite to differ with Mr. —— on the impropriety of taking arsenic with spirituous liquors, and actually employs it as a means of correcting their effects. All others that I have heard of, concur in saying it should be taken fasting.)

"As a proof of how much secrecy is observed by those who practice arsenic eating, I may mention that Dr. Arbéle says he inquired of four medical men, well acquainted with the people of the districts in question, both in the towns and country, and they could not tell him of any individual case, but knew of the custom only by report.

"Two criminal cases have been mentioned to me in which the known habit of arsenic eating was successfully pleaded in favor of the accused. The first, by Dr. Kottowitz, of Neuhaus, was that of a girl taken up in that neighborhood on strong suspicion of having poisoned one or more people with arsenic, and though circumstances were strongly against her, yet the systematic arsenic eating in the district was pleaded so successfully in her favor, that she was acquitted, and still lives near Neuhaus, but is believed by every one to be guilty. The other case was mentioned by Dr. Lorenz. A woman was accused of poisoning her husband, but brought such clear proof that he was an arsenic eater as fully to account for arsenic being found in the body. She was of course acquitted.

"One fact mentioned to me by some friends is well worthy of note. They say: 'In this part of the world when a graveyard is full, it is shut up for about twelve years, when all the graves which are not private property by purchase are dug up, the bones collected in the charnel house, the ground plowed over, and burying begins again. On these occasions the bodies of arsenic eaters are found almost unchanged and recognizable by their friends. Many people suppose that the finding of these bodies is the origin of the story of the vampire.'

"In the *Medicinischer Jahrbuch des Œster Kaiserstaates*, 1822, neue Folge, there is a report by Professor Schallgruber of the Imperial Lyceum of Grätz, of an investigation undertaken by order of government into various cases of poisoning by arsenic. After giving details of six *post-mortem* examinations, he says: 'The reason of the frequency of these sad cases appears to me to be the familiarity with arsenic which exists in our country, particularly the higher parts. There is hardly a district in Upper Styria where you will not find arsenic in at least one house under the name of hydrach. They use it for the complaints of domestic animals, to kill vermin, and as a stomachic to excite an appetite. I saw one peasant show another, on the point of a knife, how much arsenic he took daily, without which he said he could not live; the quantity I should estimate at two grains. It is said, but this I will not answer for, that in that part of the country this poison is used in making cheese; and in fact several cases of poisoning by cheese have occurred in Upper Styria—one not long since. The above-mentioned peasant states, I believe truly, that they buy the arsenic from the Tyrolese, who bring into the country spirits and other medicines, and so are the cause of much mischief.'

"This report is, I believe, mentioned in Orfila's *Toxicology*, and in one or two other works, but I have not seen it quoted myself; it is interesting as being early and official evidence of arsenic eating.

"Since I received the above information, a gentleman, who was studying at this hospital, told me that when an assistant in Lincolnshire, he knew a man who began taking arsenic for some skin disease, and gradually increased the dose to five grains daily. He said he himself supplied him with this dose daily for a long time. He wrote to the medical man with whom he was assistant, and I have been for a long time promised full particulars of the case; but beyond the fact that he took five grains of arsenic, in the form of Fowler's solution, daily, for about six years, and could never leave it off without inconvenience and a return of his old complaint, I have as yet not received them.

"I have delayed publishing these facts for some time, hoping to get information on some other points for which I have written to my friends abroad; but as considerable delay takes place in all communications with them, I have thought it better to publish at once the information I have already received.

"All the parties spoken of are people on whom the fullest reliance can be placed, and who have taken much pains to ascertain the foregoing particulars. The questions which still remain unanswered are these:—

"First. Can any official report be obtained of the trials of the two people mentioned by Drs. Kottowitz and Lorenz?

"Second. Do medical men in these districts, when using arsenic medicinally, find the same cumulative effects as we experience here? Or is there anything in the air, and mode of living, which prevents it?

"Third. Can any evidence be obtained as to how much of the arsenic taken is excreted? To show whether the body gradually becomes capable of enduring its presence, or whether it acquires the power of throwing it off. (The fact of the preservation of the bodies shows that some considerable quantity must be retained.)

"I have proposed to the gentleman who furnished me with the particulars of his own case, either to make an estimate of the arsenic contained in his own urine and fæces during twenty-four hours, or to collect the same and forward them to me that I may do so, but as yet have received no answer."—(*Pharmaceutical Journal*, May, 1860.)

II.—*Studies on Strangulation.* By DR. TARDIEU.

Dr. Tardieu, in a paper on the above subject, endeavors to give an exact and practical definition to the term strangulation, as distinguished from suffocation and hanging, so as to constitute, if the term may be used, a medico-legal species. Dr. Tardieu thus defines strangulation: "An act of violence, consisting in the direct application of constriction, either around or in front of the neck, having the effect, by preventing the passage of air, of abruptly suspending respiration and life."

Our author teaches that strangulation, as a general rule, is an act of the non-suicidal kind; it is connected either with the marks of blows or wounds of the head, showing that consciousness has been destroyed before the constriction has been applied; or it is connected with suffocation, previously brought about by occlusion of the mouth and nose; or again, it is accompanied with signs of injury on other parts of the body. It is generally committed on women or new-born children, and it may, indeed, be regarded as an act only perpetrated on persons either physically feeble or paralyzed by some antecedent injury. After pointing out the difference of signs in cases where the strangulation has been made by a cord, and by the hand, and in cases where the resistance has been little and great, Dr. Tardieu notices that the face in strangulation is tumefied, violet colored, and as if marbled.

There is also a peculiar and constant sign, viz., ecchymoses, very numerous and of extremely small dimensions, upon the face, under the conjunctiva, and in the front of the throat, and in the chest. This sign is not absolute, as it may occur in suffocation; but it is very striking. The mark of the constriction by the cord differs from the mark produced by hanging, in that it is not deep; it need not be continuous around the neck; the edge of the furrow, instead of looking like parchment, is often pale, and approaches in color the violet tint of neighboring parts; it does not present any change of texture or consistency, no thinning, and no condensation. In cases where a stick or tourniquet has been used in the tightening process, a mark of the stick will be found impressed on the parts against which it rested, while in instances where the hand has been used, the mark of the fingers is often so fairly delineated, that the hand which the murderer used, and his position to his victim, may be determined. The appearances of the internal parts of the body are deep ecchymosis of the soft parts of the neck. This is especially brought out in instances where the hand has been the compressing body. The larynx and the trachea are rarely the seat of great mischief; fracture of the cartilages and fracture or luxation of the hyoid bone are quite exceptional. The internal surface is most frequently congested and of a uniform red color; sometimes violet mucus is found in greater or lesser quantity; the mucus is sometimes sanguinolent, and, in fact, Tardieu has seen it in some cases replaced by an exudation which, having coagulated, lined the larynx.

Luxation of the spine in true strangulation is doubtful. The lungs are not often obstructed, or but little; they are of moderately uniform rose color, sometimes strongly congested, sometimes quite normal. There is almost always rupture of the superficial air-vessels, giving rise to more or less extensive emphysema. The ruptures are multiple sometimes, isolated more frequently; united in groups, they give the lung the appearance of being scattered over with pseudomembranous patches of small thickness, very white, and of various sizes, but which, on being carefully examined, are found to contain small bubbles of air, and may be caused to disappear by simple puncture. The subplural punctuated ecchymoses scattered over the surface of the lung, which are the essential signs of death by suffocation, are not found as a consequence of strangulation, but there may be apoplectic clots in the thickness of the lung tissue, together with extravasation or infiltration of blood, varying in size, but always longer and more extensive than in suffocation.

The state of the heart presents nothing essential; there is no extravasation of blood, either under the pericardium or endocardium. The heart is sometimes empty, but there is generally found a little black fluid blood.

I have only once, if ever, found some semi-coagulated blood. The brain does not present any constant or precise characters in strangulation; it is found free from change as often as congested. The state of this organ differs remarkably from what is observed after hanging, where obstruction of the vessels of the brain is as frequent as it is rare in strangulation.

The signs of incomplete or attempted strangulation are thus described: The face is swollen, violet colored, marbled, spotted with red, and livid mucus escapes from the nostrils and mouth; the eyes are bloodshot, and there is ecchymosis under the conjunctiva; the neck is swollen and painful; the voice is broken; deglutition is very painful; the swelling extends to the entire cervical region, and to the lower part of the jaw; it is diffuse and accompanied by an ecchymotic coloring of the skin. Sometimes the swelling of the face and of the neck is considerable, and these parts are of a bluish color nearly throughout. In some cases the mark of the fingers is visible—more so than in complete strangulation—for the simple reason that, while life continues, ecchymoses have time to appear, and to render the mark more and more apparent.

Incomplete strangulation may produce a loss of consciousness for some hours, but in all cases there remains for some time an obstruction in the throat, great difficulty in speaking and swallowing, and various nervous disturbances. Thus violent compression of the neck and contusions of the cellular tissue and muscles may produce inflammation and even abscess.—(*Ann. d'Hygiène publique*, tome xi. p. 106, and *British and Foreign Medico-Chirurgical Review*, April, 1860.)

